# Usability and acceptability of a cognitive training intervention (SMART) for people with multiple sclerosis (MS): A prefeasibility formative evaluation

**DOI:** 10.3310/nihropenres.13274.1

**Published:** 2022-05-23

**Authors:** Alexandra C. Frost, Nima Golijani-Moghaddam, Rupert Burge, David L. Dawson, Nikos Evangelou, Bryan Roche, James Turton, Annie Hawton, Graham Law, Elise Rowan, Roshan das Nair

**Affiliations:** 1 Institute of Mental Health, Innovation Park, Triumph Rd, Nottingham, NG7 2TU, UK; 2 University of Lincoln, Sarah Swift Building, Brayford Pool, Lincoln, LN6 7TS, UK; 3 School of Medicine, University of Nottingham, C floor, South Block, Queen's Medical Centre, Nottingham, NG7 2UH, UK; 4 Department Psychology, Maynooth University, Mariavilla, Maynooth, W23 F2H6, Ireland; 5 Health Economics Group, University of Exeter Medical School, University of Exeter, Magdalen Road, Exeter, EX1 2LU, UK; 6 Lincoln Clinical Trials Unit (LinCTU), Community and Health Research Unit, School of Health and Social Care, University of Lincoln, Brayford Pool, Lincoln, LN6 7TS, UK

**Keywords:** Multiple sclerosis, cognitive rehabilitation, feasibility randomised controlled trial, relational training, usability, acceptability

## Abstract

**Background:**

Multiple sclerosis (MS) is a chronic autoimmune, inflammatory neurological disease of the central nervous system (CNS), increasing in incidence and prevalence across both developed and developing countries. Cognitive difficulties are common in MS sufferers with 70% experiencing difficulties in higher-level brain functioning such as planning, attention, problem solving, and memory. Computerised cognitive training programmes may hold promise as a treatment option for improving cognitive function in people with MS, subject to exploring and addressing potential barriers to usability and acceptability.

**Methods:**

This study aimed to test the usability and acceptability of a computerised cognitive training intervention—Strengthening Mental Abilities Through Relational Training (SMART) —for people with MS, through a mostly qualitative prefeasibility design ( *n*= 12). There were two phases of testing: (1) initial usability testing via a think-aloud protocol ( *n*= 6) and (2) alpha-testing to assess experienced acceptability over a four-week period of engagement ( *n*= 6). Data from the two phases were subjected to Framework Analysis, wherein we deductively applied the Health IT Usability Evaluation Model and Theoretical Framework of Acceptability to assess usability and acceptability, respectively.

**Results:**

Results show SMART to have satisfactory usability with participants reacting positively to the formatting, visuality, and process of the interface. Minor suggestions were made on how best to adapt SMART for people with MS, but the programme and facilitative support were generally perceived to be acceptable, with participants expressing positive feelings about taking part in the intervention, despite associated burdens.

**Conclusions:**

This prefeasibility study provides preliminary evidence of the usability and acceptability of SMART as a computerised cognitive training programme for people with MS. We conclude that we can now move forward with a feasibility trial of SMART, with the intention of proceeding to a definitive trial with cost-effectiveness analysis.

## Plain English summary

Aims: We are developing a new ‘brain training’ treatment to help people with multiple sclerosis (MS) who have problems with thinking skills ( *e.g.*, problem-solving, attention, and memory). This study aimed to test whether the training (called ‘Strengthening Mental Abilities Through Relational Training’ [‘SMART’]) is suitable for people with MS. Specifically, we assessed whether SMART was easy to use and acceptable for use in their everyday lives.

Background: MS is a long-term condition that affects the nervous system, with the number of cases increasing in both developed and developing countries. MS affects an individual’s thinking skills, which can affect their ability to go about their everyday lives. Brain training has potential for improving thinking skills in people with MS, provided ease of use and factors impacting willingness to use the training are explored.

Design and methods used: This study used a mix of methods, such as scores from objective tests and verbal feedback, to explore whether SMART is easy to use and acceptable for people with MS. The study had two phases: think-aloud interviews (participants provided feedback on whether the training interface and guidance were easy to use) and then the alpha-testing phase (participants tested the training over time, and then gave feedback on acceptability). Common and salient themes were identified in both phases.

Results: Participants found SMART to be suitably easy to use and acceptable for use by people with MS. Participants thought that the interface was visually appealing, and easy to operate and navigate. Participants made minor suggestions for improving the intervention, but feedback was generally positive, despite demands on time and energy.

Discussion: SMART appears to be suitable for people with MS. We conclude that we can go ahead with the next phase of testing SMART, as a possible treatment for improving thinking skills in people with MS.

## Introduction

Multiple sclerosis (MS) is a chronic autoimmune, inflammatory neurological disease of the central nervous system (CNS) ( Calabresi, 2004). MS affects one in every 600 people in the UK, with 130 new diagnoses weekly ( MS Trust, 2020). MS typically presents itself in adults aged 20 to 45 years old; infrequently, it can arise in childhood or late middle age ( Cree, 2007). MS has a diverse and unpredictable range of physical and psychological effects such as spasticity, fatigue, cognitive impairments, depression, bowel dysfunction, bladder dysfunction, and pain ( Crayton & Rossman, 2006).

Cognitive impairment has been recognised as an important characteristic of MS ( Amato *et al*., 2006), with 70% of people experiencing difficulties in higher-level brain functioning such as problem solving, memory, attention, and planning ( Chiaravalloti & DeLuca, 2008). These cognitive difficulties have been recognised as the most debilitating and distressing sequalae of MS ( Dorning *et al*., 2013). Cognitive difficulties have profound effects on everyday functioning—for example, one’s ability to work, drive, and maintain healthy interpersonal relationships ( Amato *et al*., 2001; Rao *et al*., 1991; Schultheis *et al*., 2001)—impacting quality of life (QoL) ( Patti, 2009) with potentially detrimental implications for perceived competence and self-worth ( European Multiple Sclerosis Platform, 2012). Furthermore, treatment consisting of cognitive rehabilitation is not commonly offered through the National Health Service (NHS) ( Klein *et al*., 2019a), despite patients being receptive to treatment and perceiving it as beneficial ( das Nair *et al*., 2015; Klein *et al*., 2019b). There is therefore an apparent need for further research centring on developing interventions and treatment strategies for cognitive impairments in MS ( Patti, 2009).

Computerised cognitive training interventions have demonstrated positive effects on some indicators of cognitive performance for people with MS ( Cicerone *et al*., 2011; Goverover *et al*., 2018; Mitolo *et al*., 2015; O’Brien *et al*., 2008), but with limited evidence for generalised transfer to everyday functional outcomes and weak understanding of how to optimise the design of these interventions ( Lampit *et al*., 2019). We propose to examine a novel computerised cognitive training programme—Strengthening Mental Abilities through Relational Training (SMART)—which holds advantages over previous approaches building on multiple exemplar training ( Cassidy *et al*., 2011). Previous interventions are often atheoretical and delivered as part of a broader package of support, obfuscating understanding of active components. Unlike other offerings, SMART was developed from a well-established theory of cognition and language. Additionally, SMART narrowly targets a specific set of instrumental cognitive behaviours that are designed to generalise to everyday functioning.

### The SMART programme

The SMART programme is a web-based computerised cognitive training programme that directly trains ‘relational skills’ (abilities to flexibly relate concepts to one another) that are critical to information processing and sense-making ( Cassidy *et al*., 2016). SMART is a multiple-exemplar-based training programme embedded in behavioural science; specifically, Relational Frame Theory (RFT) ( Dymond & Roche, 2013; Hayes *et al*., 2001).

 RFT strives to systematise a diverse variety of cognitive skills apropos a smaller array of underlying teachable skills, known as Relational Framing Skills. These framing skills are also considered to be marginally associated with the more commonly known Relational Reasoning Skills ( *e.g.*, Halford *et al*., 2010). These skills are developed over time and are influenced by environmental factors ( Barnes-Holmes *et al*., 2001). Their development supports cognitive abilities such as language, deductive reasoning, and problem solving ( Cassidy *et al*., 2016; Cassidy *et al*., 2011). By targeting the key components of cognition, it can be theorised that SMART can promote improved cognitive functioning across multiple domains ( Cassidy *et al*., 2016). There is a growing evidence base to support this proposition.

Previous research using an improved multiple-exemplar-based relational frame training intervention, found statistically significant improvements in full-scale IQ, in children with a range of educational and behavioural difficulties ( Cassidy *et al*., 2011). Another study focussing on relational frame theory, mathematical, and logistical skills using a multi-exemplar intervention aimed at enhancing intellectual performance, found that an experimental group using SMART performed significantly better than the control group on tasks of standard progressive matrices ( Thirus *et al*., 2016). Supporting the theory that interventions grounded in behavioural science research can improve IQ measurements and can have more ecological outcomes in scholastic aptitude, *e.g.*, maths and reading assessments.

 Notwithstanding preliminary studies in patients with Alzheimer’s disease ( Presti *et al*., 2018) and cerebral palsy ( Wotherspoon *et al*., 2019), there is limited data on the suitability of SMART as a clinical intervention. SMART is a potentially promising intervention for improving cognitive abilities in MS, but its usability and acceptability for people with MS is untested. It is critical to consider and evaluate the usability and acceptability of online tools, to ensure accessibility and potential for beneficial uptake and adherence ( Christie *et al*., 2018; Eicher *et al*., 2019; Rodrigues *et al*., 2018).

The benefits of completing interventions at home have been highlighted in the context of the COVID-19 global pandemic ( Mantovani *et al*., 2020; Shaw *et al*., 2021) wherein remotely accessible interventions have enabled continuity of care. Additionally, these benefits will extend beyond current circumstance as many people with MS struggle with mobility and attending clinics, which may be further impeded by where they live ( Mantovani *et al*., 2020). There is a need to optimise the user experience of remote computerised interventions, to potentiate successful implementation.

Thus, this study aimed to assess the usability and acceptability of SMART as a computerised cognitive training intervention for people with MS, through a mixed-methods design: a primarily qualitative design with embedded quantitative data to enrich sample description and support some of the judgements surrounding usability and acceptability ( *i.e.*, a concurrent nested design; Clark *et al*., 2008). The study was a prefeasibility study, conducted to evaluate and optimise the SMART intervention ahead of a prospective feasibility trial.

## Methods

This was a prefeasibility study using a mixed-methods design to assess the usability and acceptability of SMART for people with MS that was conducted ahead of a prospective feasibility trial (ClinicalTrials.gov, NCT04975685; registered on July 23, 2021). A completed COREQ checklist, interview guides, questionnaires, consent forms and debrief information sheets used in this study can be found as *Extended data* ( Frost *et al*., 2022).

### Ethical approval and consent

Approval was granted by the University of Lincoln Human Ethics Committee on 14 ^th^ May 2021 (UoLReview Reference 2021_6692). All participants gave full informed written consent and the right to withdraw before participating in this study and in a debrief post-study completion. Data were stored on a password protected OneDrive associated with the University of Lincoln.

### Participants

Participants were recruited via three routes:

1. The Nottingham-based MS Patient and Public Involvement group supporting the larger NIHR-funded SMART MS project, of which this study is on (preliminary component).
2. Social media platforms of MS groups (including charitable organisations such as the MS Society, MS Trust, and MS UK) with chain referral of shared study invitations.
3. Snowball sampling of existing participants. Participants involved in social media MS support groups were encouraged to share the study information sheet and contact information with potential participants.

To be eligible for inclusion, individuals were required to (1) have an MS diagnosis received ≥3-months prior to study enrolment, (2) be aged between 18 and 89 years old (to meet standardisation criteria of psychometric assessments), (3) be able to read and speak English, (4) have access to a computer/tablet/smartphone, and (5) be able to give informed consent.

We recruited two groups of six participants (one group for each phase of testing). Evidence has shown data from five participants identified 80% of usability issues ( Silva *et al*., 2021). Therefore, six participants were deemed to be sufficient to identify key SMART-usability and acceptability issues in the MS population.

### Data collection

Participants gave e-consent, which was fully integrated into the questionnaire. Participants were informed of their right to withdraw in the information sheet and debrief sheet and were tracked using a participant number given upon consent. A total of 100% retention of participants was noted for this phase of testing. Both the think-aloud protocol and alpha-testing involved interviews. These were chosen to get as much feedback from participants as possible. Interviews took place online over video call. All interview transcripts were kept securely stored on a password protected OneDrive associated with the University of Lincoln. No follow-ups were required.

***Think-aloud usability protocol.*** Data-collection for the think-aloud protocol began June 8th, 2021. Think-aloud protocols are commonly employed in usability testing ( Geiselman *et al*., 1984; Lewis, 1982) and we used this method to assess how participants understand and respond to standard SMART instructions and procedures, whilst interacting with the software, a full description of the SMART intervention is available as *Extended data* ( Frost *et al*., 2022), following the Template for Intervention Description and Replication (TIDieR) checklist. For the think-aloud protocol, an interview guide was created consisting of 12 concurrent ‘think-aloud’ feedback prompts, supplemented by six retrospective prompts. Think-aloud prompts consisted of questions such as “What are you thinking as you look at this?”, and “What is your first impression of this screen/interface?”. Retrospective prompts included “Was anything surprising or did not perform as expected?”, and “What could we do to make this programme more user friendly for people with MS?”. Before guiding participants through the think-aloud protocol with the SMART interface, a training task (taken from Willis, 2005) was used to orient participants to the think-aloud method. Participants were encouraged to “Visualise the place where you live and think about how many windows there are in that place. As you count up the windows, tell me what you are seeing and thinking about”. This task encouraged verbalisation of participants’ thought processes

***Alpha-testing.*** Data-collection for alpha-testing began August 16th, 2021. Alpha-testing is a form of pretesting to assess acceptability under real-life conditions, ideally conducted with potential end-users of an intervention. Alpha-testing is a step used in systematic processes for developing patient-facing resources, such as decision aids ( Coulter *et al*., 2013). In our study, Alpha-testing participants engaged with the SMART programme independently over the course of four weeks, and were advised to perform 90 minutes per week, in blocks of three (preferably as 3*30-minute sessions). Participants had weekly check-in support calls with a researcher: these calls served to enable participant engagement with the SMART programme ( *e.g.*, addressing arising queries) whilst simultaneously gathering data on problems encountered during the testing phase. At the end of the four-week period, participants were then interviewed about their experiences, with a focus on evaluating the SMART programme and adjunctive support calls. Alpha-test questions were generated consisting of 12 questions about the SMART programme ( *e.g.*, “Were there any times when you had a bad experience with the programme? What happened?”), and six questions regarding the support calls ( *e.g.*, feedback on the support calls was useful for planning future implementation of SMART ( *i.e.*, whether support calls should be provided alongside the training, and what will be the likely focus and function of these calls [based on topics commonly addressed in the alpha-testing]).

***Measures.***

**Symbol Digit Modalities Test (SDMT) ( Smith, 1982)**

To gauge cognitive abilities in the study samples, all participants were asked to do the SDMT (oral version). The SDMT is a symbol substitution test, examining processing speed and attention, and is considered the most sensitive single screening test for cognitive impairment in MS ( Benedict *et al*., 2017). Participants are asked to match the symbol to the corresponding number, shown in a diagram at the top of the page. Participants are given 90 seconds to correctly correspond as many symbols to the numbers as possible. The SDMT has good test-retest reliability (r= 0.97; Benedict *et al*., 2017; Smith, 1982).

**Perceived Deficits Questionnaire (PDQ) ( Sullivan *et al*., 1990)**

To characterise subjective cognitive difficulties in the study samples, all participants completed the 20–item PDQ: assessing their cognitive functioning within the past four weeks. The PDQ yields a total score ranging from 0–80, with higher scores indicating greater perceived impairment. The PDQ has good internal consistency (ɑ= 0.93; Sullivan *et al*., 1990).

### Procedure

***Think-aloud protocol.*** Participants were contacted via the SMART study email address and sent a link to a Microsoft Teams call. Participants were presented with the information sheet detailing important information about the study, followed by a consent form; materials were presented and completed online via a survey platform Qualtrics (Qualtrics Survey Platform, RRID:SCR_016728; free alternative is Google Docs). If consent criteria were met participants were asked to share their screen and login to the SMART programme using their specific login details that were sent previously via email. Once logged in participants were taken through the opening screen and different pages of the interface, whilst using the ‘think-aloud’ method to generate feedback on the programme. Following on from this, participants went through the pre-assessment stage, followed by two of the module stages of SMART. Participants were encouraged to use ‘think-aloud’ throughout the process. Participants then looked through the SMART report page of the interface to generate feedback. Participants were asked cognitive interview questions regarding the interface from a set interview guide.

Participants were asked to perform the SDMT, and the procedure ended with the PDQ. Both measures had previously been sent by email prior to participation. The SDMT as a word document attachment and the PDQ as a Qualtrics link to the web-based questionnaire.

***Alpha-testing.*** Participants were sent a link to a Microsoft Teams meeting via the SMART study email address after registering their interest in the study. Participants were sent links to an information sheet, via Qualtrics, a link to a Qualtrics consent form, login details for SMART, an SDMT attachment, and the Qualtrics link to the PDQ. Participants first read the information sheet and then read and signed the consent form. Next, participants were taken through the interface briefly to ensure they understood how to navigate the programme. Following on from this, participants performed the SDMT, and the PDQ. Participants were asked to use the SMART programme, advising them to use it three times a week for 30 minutes each. Weekly online check-ins with participants were organised by email and participants were sent a Microsoft Teams link for the check-in with the SMART team researcher. Check-ins addressed any queries and problems that may have arisen. A final interview was hosted on the fourth week check-in with the set alpha-testing questions.

### Data-analysis

Our research was conducted from a critical-realist position (combining realist ontology with relativist epistemology; McEvoy & Richards, 2003), which recognises the fallibility and context-dependency of our observations as representations of the underlying reality. This position informed our use of multiple theory-driven methods and study phases (with a primary focus on contextually sensitive qualitative data) to achieve our evaluative aims and our use of team-wide quality assurance processes (offsetting the limited view of any single analyst through triangulation of multiple researcher perspectives). For each phase of testing, we analysed data using a five-step Framework Analysis procedure ( Gale *et al*., 2013): transcription, familiarisation, coding, developing an analytical framework, and applying the framework. We took a primarily deductive approach to Framework Analysis, such that the analytical framework was informed by a priori theory, whilst allowing for secondary identification of data-driven themes. Transcripts were primarily coded by Alexandra Frost using NVivo software, version 12 (RRID:SCR_014802; free alternative is Taguette) and secondarily reviewed by NGM for concordance; identified discrepancies were discussed and resolved. The remaining co-authors reviewed the final written analyses in terms of credibility, dependability, confirmability, and transferability ( Stenfors *et al*., 2020). For data from the think-aloud protocol, analysis focussed on experiences of usability during initial interactions with SMART, and we applied the Health IT Usability Evaluation Model (Health-ITUEM; Brown *et al*., 2013) to pre-select eight usability themes: error prevention, completeness, memorability, information needs, flexibility/customisability, learnability, performance speed, and competency. For data from alpha-testing, analysis focussed on experienced acceptability over a sustained (four-week) period of engagement with the SMART intervention and we applied the Theoretical Framework of Acceptability (TFA; Sekhon *et al*., 2017) to pre-select seven acceptability themes: affective attitude, perceived effectiveness, burden, ethicality, opportunity costs, intervention coherence, and self-efficacy. Recognising the brevity of the alpha-testing period, we examined ‘perceived effectiveness’ as a subcomponent of ‘intervention coherence’: *i.e.*, that participants may have an early impression of any changes/intervention effects, but that these would likely inform their sense of the intervention’s internal consistency (that it seems to be working as intended). Quantitative data were analysed using IBM SPSS Statistics (RRID:SCR_016479; free alternative is PSPP).

## Results

### Participant characteristics: demographics and cognitive assessment results

***Think-aloud protocol participants.*** Six participants, five women (83.3%) and one man (16.7%) took part in this phase of testing. Participant age ranged from 38 to 63 years old (M= 48.67, SD= 9.94); half had a diagnosis of secondary-progressive MS and half had relapsing-remitting MS. SDMT scores ranged from 38 to 51 (M= 48.00, SD= 8.37) with three participants in the impaired range ( *i.e.*, scores at least 1.5 SDs below the age- and education-adjusted normative average; Strober *et al*., 2020). Total scores for the PDQ ranged between 28 and 65 (M= 54.33, SD= 13.89) with higher scores indicating higher perceived deficits.

***Alpha-testing participants.*** Four women (66.7%) and two men (33.3%) took part in this phase of the research. Participants ranged in age from 31 to 56 years old (M= 43.34, SD= 9.34); half had a diagnosis of secondary-progressive MS and half had relapsing-remitting MS. SDMT scores ranged from 27 to 63 (M= 48.83, SD= 12.79) with two participants in the impaired range. Total scores for the PDQ ranged between 45 and 68 (M= 56.17, SD= 8.98). The sample characteristics and cognitive assessment results are publicly available as *Underlying data* ( Frost *et al*., 2022).

### Think-aloud protocol results

Data from the think-aloud protocol interviews were deductively mapped to eight usability domains in the Health-ITUEM; additionally, two usability-relevant themes were inductively identified from the data. Findings are summarised below with supporting quotes provided in Table 1.

**Table 1.** Usability of SMART for people with MS.

Usability domain	Description	Quotes illustrating perceived strengths (+) and possible problems (-) in this domain
**Error prevention**	System offers error management, such as error messages as feedback, error correction through undo function, or error prevention, such as instructions or reminders, to assist users performing tasks	• “I didn’t know what the bleep signified… should I try and answer a question wrong… will it make a different noise?” (P3 _TAP_). (-) • “The beeping when you press the answer… I think I prefer the beeping before” (P4TAP). (+) • “And also, whilst I am doing them … it doesn’t give you any feedback on whether you’ve got them right or wrong” (P6 _TAP_). (-)
**Completeness**	System is able to assist users to successfully complete tasks.	All participants (six of six) were able to successfully navigate the interface, complete the practice stages, and proceed through the two test blocks as per protocol.
**Memorability**	Users can remember easily how to perform tasks through the system	• “It’s probably quite easy but I just can’t remember those kinds of things. That’s the problem…” (P1 _TAP_) (-) • “You didn’t have to store… You got the facts and the question [displayed together onscreen], so that was really straightforward” (P6 _TAP_). (+)
**Information needs**	The information content offered by the system for basic task performance, or to improve task performance ( Amith *et al*., 2012; Norman, 2002).	• “The writing was quite clear, I think it's very, very easy. I mean, it's just a simple text” (P3 _TAP_). (+) • “Simple statements so that's still quite easy to follow” (P4TAP). (+) • “I don’t know what ‘toggle nav’ means” (P2 _TAP_) (-) • “Instructions were clear enough” (P6 _TAP_). (+)
**Flexibility/customisability**	System provides more than one way to accomplish tasks, which allows users to operate system as preferred	• “Just for those that can’t use their hands, they would probably need to go to do that with voice instead” (P1 _TAP_). (-) • “I have friends … they would struggle … they have things for blowing up their screens … making it easier for them to see visually… a friend of mine on his phone … has colours inverted because he finds it easier to see” (P3 _TAP_). (-) • “Making the writing a bit bigger … For people who struggle to see things” (P4TAP). (-) • “I think could be accessed by anyone easily” (P6 _TAP_). (+)
**Learnability**	Users are able to easily learn how to operate the system	• “Very clear and easy to understand” (P1 _TAP_). (+) • “My brain was thinking this is really hard, but as you got into it, it actually got easier” (P2 _TAP_). (+) • “Feel like it was quite easy to understand… Especially 'cause they start off with simple statements… And then obviously they build. So yeah, no. It kind of prepares you.” (P4TAP). (+) • “Buttons were very clear… green and blue …an immediate recognition thing” (P4TAP). (+) • “Because I’ve never done anything like that before … I didn’t really know what I should do … and I found it difficult” (P5 _TAP_). (-) • “The more you did it, the easier it would become” (P6 _TAP_). (+)
**Performance speed**	Users are able use the system efficiently	• “Easy enough to navigate” (P1 _TAP_). (+) • “I pressed the wrong button so yeah did one wrong… It’s very easy to press the wrong button” (P2 _TAP_). (-) • “I think it flowed really, really well” (P2 _TAP_). (+) • “It looks quite easy to navigate” (P5 _TAP_). (+)
**Competency**	Users are confident in their ability to perform tasks using the system, based on Social Cognitive Theory ( Burnay *et al*., 2013; Luxton *et al*., 2012).	• “Yeah, I mean the way to do it was easy to understand. Obviously, the questions themselves took some definite thought.” (P2 _TAP_). (+) • “It’s very clear: X is opposite to Y… very, very straightforward, unless I’m missing something” (P6 _TAP_). (+) • “It’s sort of user friendly and then I’m so keen to do a bit more and sort of build on what I’ve started so far” (P3 _TAP_). (+)
**Other outcomes**	*Visual clarity and aesthetics*	• “It’s clear and it looks welcoming” (P1 _TAP_). (+) • “I can’t see how you’d improve it” (P6 _TAP_). (+) • “Looks so clean and not so, not too cluttered or anything” (P3 _TAP_). (+)
	*Enjoyment*	• “So that was fun. Enjoyed that” (P1 _TAP_). (+) • “I quite enjoyed that” (P2 _TAP_). (+) • “It’s quite enjoyable” (P4TAP). (+) • “Yep. Enjoy that, yeah, brilliant.” (P5 _TAP_). (+) • “The thing was fun” (P3 _TAP_). (+)

*Note.* (+) = Usability factor positively present. (-) = Usability factor problematic/absent. MS= Multiple sclerosis. P *x* = Participant identification code. TAP = Think-aloud protocol. SMART = Strengthening Mental Abilities through Relational Training.

***Error prevention.*** Participants had mixed and changing views about the use of audio cues to provide feedback on task performance during practice trials. Some struggled initially to discriminate cues for correct vs. incorrect responses and one participant did not have speakers enabled at first so missed these cues. After progressing to test trials (where feedback is no longer provided) participants commented on the loss of feedback and retrospectively indicated that the audio cues had been helpful to support early practice.

***Completeness.*** The SMART programme supported all participants to successfully complete assigned training tasks.

***Memorability.*** Few references were made to this domain (likely reflecting the short period of engagement with SMART during initial usability testing). One participant observed that SMART presented sufficient onscreen information to guide current performance ( *i.e.*, did not place demands on participants to retain information over time). One participant referred to the demands on working memory (when cross-referencing multiple stimuli in a task) though acknowledged that this challenge was part of the training: “I mean it made me think” (Participant 1, Think Aloud Protocol [P1 _TAP_]).

***Information needs.*** Participants were generally positive about information provided within SMART to instruct and guide task performance, commenting on its clarity. Participants made specific reference to the ‘tips’ provided within SMART ( *e.g.*, advising that participants refrain from making notes during tasks), indicating that these were helpful in enabling them to engage with the task without overthinking or strategizing.

***Flexibility/customisability.*** Whilst participants generally found SMART to function in ways that were accessible for them, some gave examples of people they know with MS who would benefit from adjustments to meet their needs and preferences ( *e.g.*, alternative input modalities, adaptable font size or colour-scheme). Although there was also acknowledgment that it may be difficult to pre-empt the needs of individuals with MS: “MS affects everybody differently… so there’s nothing I could particularly suggest that would you know be a benefit to anybody with MS” (P3 _TAP_).

***Learnability.*** Learnability was a noted strength of SMART, with participants commenting on the graded complexity, which enabled them to gain a sense of mastery through direct experience of completing the puzzle-based tasks. Although P5 initially commented on the difficulty of the task (during practice trials, as quoted in Table 1) their view shifted after a further block of trials: “It seems to be easier” (P5 _TAP_).

***Performance speed.*** In general, participants were able to efficiently navigate the system and progress fluently through the planned tasks. One noted issue with SMART-use was around the potential to accidentally press the wrong response option—particularly when using a smaller device (smartphone)—and this was linked to MS symptoms such as tremor and reduced hand-eye coordination.

***Competency.*** Linked to system learnability, participants expressed confidence in their ability to use SMART. By the end of the exercise, all participants indicated a sense of proficiency with the system and that they would be keen to use the system again.

***Visual clarity and aesthetics.*** Within think-aloud responses, we inductively identified a theme around the visual appeal of SMART, with participants commenting on the clear and attractive presentation of the SMART interface.

***Enjoyment.*** All participants made unprompted comments about enjoying their experience with SMART, and we identified enjoyment as a further inductive theme, with likely relevance to experiences of usability and propensity to engage with the software.

### Alpha-testing results

Table 2 presents a summary of findings (with supportive quotes) from interviews with participants who completed SMART alpha-testing (using the SMART programme over a period of four weeks, with weekly telephone support). Data from these interviews were deductively mapped to six acceptability domains in the TFA; then, within each domain, relevant subthemes were inductively identified.

**Table 2.** Acceptability of SMART in terms of Theoretical Framework of Acceptability: alpha-testing.

Domain and definition	Subthemes	Summary of findings
**Affective attitude** *How an individual feels taking part in* *an intervention (retrospective)*	**Attitudes towards the** **intervention** *Positive (+)* *Negative (-)* **Attitudes towards the** **check-in calls** *Positive (+)* *Negative (-)* *Neutral (n)* **Recommend the** **programme to others**	**Attitudes towards the intervention** *P3 _AT_: “It felt like a positive experience to start with. I liked it because I could do it when I wanted. It was relatively easy* *to get into once I got used to it” (+)* *P1 _AT_: “It was fun doing it […] wasn’t much that I didn’t like” (+)* **Attitudes towards the check-in calls** *P1 _AT_: “I liked the one to one, the check-ins and things. I asked the question, I got an answer, pretty straightforward* *straight away […] so I liked that, you would get an answer basically straight away and it would be sorted” (+)* *P2 _AT_: “I don’t know that I found anything particularly helpful or unhelpful. It was more just to check-in. So, as I didn’t* *have any particular questions or problems … did ask a few questions, so that was helpful to have that … I’m on the* *fence on this part. It was just part of the study” (n)* *P6 _AT_: “I think it was just carrying on. In encouraging, I knew that I wasn’t just being left alone to sot of do it …* *reasonably well motivated … it worked quite well the weekly check-in and sort of made me think … I need to do the* *session” (+)* *P4 _AT_: “yes, as in reminding me to use it” (+)* **Recommend the programme to others** *P1 _AT_: “it could be helpful. I would let someone know and tell them, yeah, you should do this ‘cause it will help with* *your mind and help your memory and job, doing what you’re doing, you know thinking skills”* *P3 _AT_: “Yeah, definitely, I think everybody should have a go. I wouldn’t have wanted it in the beginning. I’d say after the* *first six months perhaps, any sooner would be too soon […] they’ve got enough to cope with”* *P2 _AT_: “I don’t know because I think it’s hard to really extrapolate from what you’re doing to real life, and there was a* *part of me that thought, am I actually just being trained up to use this programme, or is it actually going to have a* *significant impact on my cognitive skills?”* *P4 _AT_: “I would yes, the people I know with MS, I don’t think it would be […] not that it wouldn’t be suitable, it’s just* *them as people would probably get a bit frustrated and not stick with it. But certainly, if I knew anyone who had* *similar cognitive decline to me then I would definitely mention it to them”*
**Burden** *The perceived amount of effort that* *is required to participate in the* *intervention*	**Tiredness** *Fatigue* **Time burdens** *Motivation* **MS symptoms** *Implications* **Frustration** *Stressful*	**Tiredness** *P3 _AT_: “too repetitive, too much of the same … lost interest”* *P3 _AT_: “when I was tired … it was more of a struggle … I tried doing it at different times of the day and … that really* *affects how you manage it”* *P4 _AT_: “the practice rounds are helpful because they’re almost a bit simpler, or the way it’s laid out … as much as I’ve* *complained about the practice things I do get why they’re there … maybe if they could be shorter, ‘cause I think* *they’re about the same length as the … main quiz … once I’ve done that and then the actual quiz, that’s quite a lot* *… especially when you get cognitive fatigue … so it’s kind of a shame that you’ve … had to use half of your energy on* *the practice”* **Time burdens** *P2 _AT_: “I think having a quiet space that I could do it where I couldn’t be interrupted was important … I wished I could* *have been more motivated to do it … it was quite difficult, which I hadn’t anticipated, but I think that’s just because* *I’ve* *had my head elsewhere on so many other things”* *P3 _AT_: “it was just making the time for it really … my husband also thought it would be beneficial … we just both* *thought that long term it will probably help me”* **MS symptoms** *P3 _AT_: “I think people obviously need to be able to operate it verbally… because sometimes our fingers are quite a* *problem”* *P4 _AT_: “email reminders … if it was like you haven’t logged in and done some time here today or you’ve not logged in* *in a few days … ‘cause my memory is awful, even with the calendar and reminders and everything else”* **Frustration** *P4 _AT_: “Sometimes it would just be like pressing the wrong thing …. [It would be good to have] a choice of not doing* *the practice one again … it’s really frustrating”* *P5 _AT_: “Being frustrated because they got one wrong and went back to the start … quite demotivating really … but I* *kept going because I knew I was doing the trial but yes, if it wasn’t in the trial, I think I would have just sacked it off”* *P3 _AT_ “There are elements where I found it sometimes a bit mundane … I felt frustrated … it felt too much of the same”*
**Ethicality – Values** *The extent to which the intervention* *has a good fit with an individual’s* *value system*	**Perceived benefits** *Cognitive performance*	**Perceived benefits** *P6 _AT_: “I’m quite keen to keep myself because I had to stop working after I was diagnosed with MS. I want to keep my* *brain active, so doing something like this, I’ve really enjoyed because it has made me think a bit more than just my* *day-to-day living”* *P4 _AT_: “I don’t work, that helps, so if I need something to do, especially using your brain, the fact that I retired is* *probably the main thing … I’ve got my projects to do”*
**Intervention coherence** *The extent to which the participant* *understands the intervention and* *how it works*	**Credibility** *Perceived effectiveness* *Face validity* **Understanding of the** **interface** *Formatting* *Clarity* **Progression of the** **intervention** *Difficulty of tasks* *Learning curve*	**Credibility** *P4 _AT_: “I like the graduation of the levels, like I said, this week, it really kind of clicked with me, kind of where I didn’t* *have to think, it all was clicking into place, I felt like my brain was ticking over a bit more, like it used to a long time* *ago”* *P3 _AT_: “I liked it because I felt like it must be doing me some good. I found it quite easy, and I think the benefits will be* *huge for something like this. I think the scope for making it even bigger and better is also huge, so I think it’s a really* *good thing, really well received”* *P4 _AT_: “I have always done like brain training, type things like Nintendo brain training […] analytical type of stuff […]* *but nothing like this, this is completely new to what I’ve experienced”* *P6 _AT_: “Specifically, I mean I’ve used brain sort of training apps on my phone and stuff before, but they certainly* *don’t. I’ve never really seen the way the questions were asked [the format of SMART was] always the same, although* *the parameters changed, it was always set out in the same way, which was fine. I didn’t think it was like boring or* *anything like that. I’ve not seen it done in that way before”* **Understanding of the interface** *P3 _AT_: “Initially I was getting the personal login, then I seemed to lose my way a bit. I don’t quite know what I did* *wrong. So, it was me rather than the system, I just couldn’t remember how to get into it. Once I’d asked [facilitator]* *to remind me how to do that then that was fine, so I didn’t really have a problem with the system, I found it very easy* *to use”* *P4 _AT_: “I really like the programme. Actually, I think it’s simple […] I think it’s laid out well. It’s obvious when you look at* *it, what you’re supposed to do”* *P6 _AT_: “I mean in terms of the actual use of the programme, there wasn’t sort of really anything too bad with it. My MS* *affects my hands. So, the fact that it was just sort of, pressing the mouse rather than having to type anything, it was* *great for me, so I can do that without causing myself too much discomfort”* *P2 _AT_: “I don’t think I had any problems. It was relatively straightforward. Logging in was easy. Going back to the* *homepage was easy. It was really user friendly I thought”* **Progression of the intervention** *P5 _AT_: “when I got them all right, made me feel quite good really […] some levels I just get stuck, and I couldn’t seem* *to get past them. I was struggling to see the patterns broadly”* *P6 _AT_: “I think the way the questions were worded was quite good […] I’ve actually found it quite easy to do some of* *the sections which were meant to be, by the look of it, harder”*
**Opportunity costs** *The extent to which benefits, profits,* *or values must be given up to engage* *in the intervention*	**Time costs** *Loss of personal time* **Family costs** *Loss of time with family* *Caring arrangements* *Fitting around family* *schedule* **Work arrangements** *Fitting around work* *schedule* *Retirement*	**Time costs** *P1 _AT_: “the hardest part was trying to do it in a free time. Obviously being a single father, trying to find the time to do* *it”* *P5 _AT_: “Only for my personal situation of having to go to school – time scheduling”* *P4 _AT_: “The only thing I don’t like about it is when you do it wrong, especially on the practice, it sends you back. So,* *you have to do it all again. If you get one wrong in the actual test itself, you then have to go back and redo the* *preparation. It feels a bit long winded and that gets a bit boring then”* *P2 _AT_: “I think I would like to be able to see progress, so I would like to be able to see how far through a block. So, I* *think having that progressive thing would have been helpful”* *P6 _AT_: “The fact that you had to get every single question right: I can kind of understand the point of the training base* *is to make sure you understand it. But it’s quite frustrating if you got one wrong towards the end and had to go all* *the way back through again”* **Family costs** *P1 _AT_: “Family arrangements. ‘cause then if I needed someone else to look after the littlun and just spare like 5-10* *minutes”* P5 _AT_: “Timings I guess, for me and the children being out of school, the day gave me free time to just do it when I was on my own. I didn’t tend to do it at weekends, it was more during the week. So that made it more possible for me” **Work arrangements** *P2 _AT_: “Having to fit it in around all my other work, which is a lot is online and actually then to have to go back online I* *found quite frustrating”* *P4 _AT_: “I don’t work, that helps, so if I need something to do, especially using your brain, the fact that I retired is* *probably the main thing”*
**Self-efficacy** *The participants confidence that* *they can perform the behaviour(s)* *required to participate in the* *intervention*	**Mastery** *Perceived ability to use the* *programme* *Patterns arising* *Perceived improvement in* *cognitive function*	**Mastery** *P5 _AT_: “When I got them all right, made me feel quite good really […] some levels I just get stuck, and I couldn’t seem* *to get past them. I was struggling to see the patterns broadly”* *P2 _AT_: “It was systematic, you kind of knew what to expect […] after you do the practice, you have a sense of the* *specific things it’s dealing with […] you started to see patterns that felt like you were getting better at it, so I quite like* *that”* *P5 _AT_: “I guess I don’t know how I would’ve dealt with it completely on my own. You went through it with me at the* *start. Maybe if there was – how you took me through it – there was some page like that at the start, because there* *wasn’t anything, but I knew how to use it because you showed me […] maybe for the people that were fresh to it”*

*Note.* P *x* = Participant identification code. AT = Alpha-testing. SMART = Strengthening Mental Abilities through Relational Training.

***Affective attitude.*** Affective attitude was split into two subthemes: attitudes towards the intervention (positive, negative, and neutral), and attitudes towards the check-in calls. Participants expressed positive attitudes towards the intervention, voicing a general appreciation of the tests and a subsequent enjoyment of the interface: “I did like the tests […] it was fun doing it, there wasn’t much that I didn’t like” (P1 _AT_).

Five of six participants perceived the check-in calls to aid in their ability to use the programme, claiming to benefit from the one-to-one guidance and support, whilst also utilising the meetings as an opportunity to query and as a reminder to complete the sessions. One participant displayed a neutral response to the check-in calls, indicating that they would be more useful for those who were struggling with the intervention.

When considering whether participants would recommend the programme to others, most indicated that they would, though sometimes with caveats (according to whether it would be a good fit for the person, or their stage of the MS journey). One participant wanted to see evidence of the real word impact of the training before they would recommend to others and wondered whether progression on the programme would transfer to everyday cognition.

***Burden.*** Burden was separated into four subthemes: *tiredness*, *time burdens*, *MS symptoms*, and *frustration*. Participants frequently displayed tiredness tendencies throughout the alpha-testing phase, with some attributing this to the repetitive nature of the interface and others assigning blame to the length of the exercises.

Time burdens were a recurring subtheme that seemed to negatively impact participants’ motivation. Participants discussed the difficulties of making time for the programme, and during check-in calls admitted to not using the programme as much as they would like: “It was quite difficult, which I hadn’t anticipated, but I think that’s just because I’ve had my head elsewhere on so many other things” (P2 _AT_).

MS symptoms emerged as one of the most salient themes throughout the alpha-testing. Participants experienced difficulties with hand-eye coordination, memory, and tremors, resulting in a perceived poorer performance on cognitive tasks. Participants also suggested prevention methods for memory-related MS symptoms, by altering how instructions are displayed so that cues are consistently available: “not just say it once but if it says it every time […] because obviously forgetful memories” (P1 _AT_).

Frustration was identified as the final subtheme of burden, sometimes linked to repetitiveness of the tasks and sometimes to how the system penalised errors (“being frustrated because they got one wrong and went back to the start” [P5 _AT_]), particularly given that these errors might be accidental/precipitated by MS symptoms rather than cognitive abilities.

***Ethicality – Values.*** In terms of fit with personal values, one salient report identified SMART as congruent with a participant’s priorities around maintaining cognitive health: “I want to keep my brain active, so doing something like this, I’ve really enjoyed because it has made me think a bit more than just my day-to-day living” (P6 _AT_).

***Intervention coherence.*** Intervention coherence was divided into three subthemes: *credibility*, *understanding of the interface*, and *progression of the intervention*. Participants believed the intervention to be credible as they perceived clear cognitive improvements throughout their training (“I felt like my brain was ticking over a bit more, like it used to a long time ago” [P4 _AT_]). Furthermore, participants saw not only the immediate benefit but the opportunity to expand the intervention to have even more of an influence (“I think the benefits will be huge for something like this. I think the scope for making it even bigger and better is also huge” [P3 _AT_]).

Understanding of the interface was a key subtheme, focussing on the formatting and clarity of the intervention. Most participants thought that the interface was very user-friendly, with one participant highlighting its usability for people with MS. However, one aspect became particularly salient to one participant: they had problems with logging into the interface, as remembering a new username/password combination proved difficult.

Progression of the intervention was identified as a subtheme reflecting perceptions of the intervention as suitably staged and attuned to incremental learning and performance. Participants varied, with some finding the intervention less challenging and others struggling to progress/experiencing a spike in difficulty at certain levels.

***Opportunity costs.*** Opportunity costs was split into three core subthemes: *time costs*, *family costs*, and *work arrangements*. Time costs was a prominent subtheme due to the longitudinal nature of the prefeasibility testing. Participants agreed that the most extensive time cost was the forced repetition of tasks after making errors. Working around family arrangements and caring responsibilities was salient to some individuals, who expressed the difficulty in finding space between day-to-day routines. Work demands affected participants’ motivation and ability to perform the tasks. Some participants identified being retired from work as a key factor enabling them to fully utilise the interface.

***Self-efficacy.*** Most participants were confident in their ability to use the programme, claiming that patterns arose as the stages progressed, which helped with performance. However, some encountered difficulties and felt that, without the presence of the facilitator and ability to ask for help, they may not have been able to master the interface.

### Suggestions for optimising acceptability

Analysing across themed data, we identified several suggestions for optimising acceptability, varying in saliency and level of consensus. Some suggestions were salient to specific individuals; for example, enabling verbal input, working around the requirement to generate and remember new login information ( *e.g.*, ability to sign in with extant cross-platform credentials), and targeting the offer of intervention to those who have progressed beyond an initial phase of adjusting to the diagnosis of MS. Other suggestions were more broadly agreed upon by (between two to four) participants, such as: the value of regular check-in calls to prompt and sustain engagement, a facility to progress without repetition after an error, increased variety of tasks, and instructions that are consistently present/accessible when using the programme.

## Discussion

This research study aimed to assess the usability and acceptability of SMART for people with MS, to inform a prospective feasibility trial. Overall, results showed SMART to have satisfactory usability, with participants reacting positively to the formatting, visuality, and process of the interface. Minor suggestions were made regarding how best to adapt SMART for people with MS, but the programme and facilitative support were generally perceived to be acceptable, with participants expressing positive feelings about taking part in the intervention, despite associated burdens.

In terms of specific usability factors, most participants thought that the interface was visually appealing, commenting on its clarity and appearance. These aesthetic strengths are likely to support engagement with SMART. Previous research has found associations between the likelihood of individuals participating in an intervention and the visual appeal of the interface/website ( Parlove *et al*., 2004). Moreover, visual aesthetics have been shown to support the comprehensibility of training/learning materials ( Brown, 2002). Further literature suggests that individuals tend to trust online clinical resources rated more highly in visual appeal, as opposed to mistrusting those with an unprofessional design, errors, and poor visual design ( Grabner-Krauter & Kaluscha, 2003). Research considers visual appearance to be closely linked to the usability attribute of satisfaction, as visually appealing stimuli enhance positive emotions/appraisals ( Petersen *et al*., 2019). Furthermore, participants determined the colours used and layout of the interface were beneficial in aiding them in task completion. The Institute of Medicine have reported that when considering actionable content, primary recommendations included displaying content clearly, organising well and simplifying ( Stonbraker *et al*., 2018).

Some participants found the interface to be efficient to navigate. Previous research determined navigability to be a core component in usability when developing online health interventions, for example Petersen *et al*. (2019) stated “Interventions should be designed in a way that makes it easy for users to navigate in the platform, and that provide them with sufficient navigational aids to advance in the intervention and follow intervention instructions”.

Some concerns were expressed in relation to the usability factor of performance speed. Most participants were carrying out the programme from a mobile device, and this resulted in a different layout, issues with touch screen sensors, and a smaller visual display. Participants found the interface and text to be somewhat small in the mobile layout: this contributed to errors (accidentally pressing the incorrect response-option), which some linked back to symptoms of MS, such as tremors and lack of hand/eye coordination. Therefore, it is important to consider dexterity-affecting chronic disease when designing and implementing an interface. SMART offers both mobile/tablet and computer access minimising these difficulties. However, it may be worth addressing the formatting of the mobile version through further user-experience testing.

Flexibility/customisability is an important attribute when assessing usability and acceptability, and is associated with participant satisfaction, and avoidance of frustration ( Petersen *et al*., 2019). It is also loosely linked to learnability, task effectiveness, and efficiency ( Petersen *et al*., 2019). Participants identified ways in which the SMART interface could be more adaptable to accommodate MS-related difficulties (including visual impairment and dexterity), with one participant strongly believing that a facility for voice-operation would improve usability for some MS patients. In support of this suggestion, previous research with MS patients has found that they positively evaluated the usability of voice-operated smart technology ( *e.g.*, command-controlled lights, entry systems, and media; Stahl & Laub, 2017). However, research focussing on mobile technology-use by people experiencing MS-related fatigue, found that voice operation of mobile technology was only used by 6% of their sample, with some reporting difficulties using voice recognition software, due to slurred speech ( Van Kessel *et al*., 2017). Voice operation may only be needed/used by a minority of people with MS, but this facility could be highly enabling for the individuals concerned.

SMART provides extensive instructions that participants believed were clear and straightforward in supporting them to complete required tasks (error prevention). Research has highlighted the need for simple instructions and clear layouts when considering usability of specific tools ( Durand *et al*., 2012). Participants had mixed views about the auditory cues provided within SMART and identified that the interface provided minimal feedback on progress, which could be demotivating. Several studies have shown the potential value of auditory feedback in potentiating learning/progression through training ( Kluger & DeNisa, 1996; Lancini *et al*., 2020; Sweller, 1988). Conversely, feedback can have varying effects, including stunting performance ( Sapyta *et al*., 2005) such that it is important to recognise how the individual internalises and interprets feedback.

There was consensus among participants that the SMART interface was clear and readable across various devices. This is an important indication of viability for application in MS, as it is known that MS can cause significant visual impairments ( Williams, 2019). Participants suggested that the visual clarity of the SMART interface enhanced learnability, consistent with findings that interventions are easier to understand when presented using a simple design and clear communication, with reduced demands on cognitive resources ( Petersen *et al*., 2019).

Participants agreed that a degree of researcher support is helpful when using the programme. Participants felt like the support calls were a useful reminder (offsetting memory difficulties) and helped answer any queries, with some participants believing that they would have struggled to manage the training on their own. Previous research has determined that personal support can often encourage adherence to an intervention ( Petersen *et al*., 2019).

Participants identified some burdens and costs associated with (1) repetition of similar tasks and (2) stringent criteria for demonstrating learning/progressing through the SMART programme (in particular, the requirement to repeat tasks after errors). Whilst these acceptability issues are important to consider and (where possible) mitigate, the development of relational abilities requires multiple exemplar training with reinforcement of correct responding, making repetition and response-contingent progression intrinsic to learning.

Participants expressed an overall enjoyment of the interface but believed that greater variety would help to maintain engagement. Previous research suggests that it is key to acknowledge and address motivational difficulties and cognitive strain when running an intervention with specific user-groups ( Engdahl *et al*., 2021). By contrast, research has found that the more options and actions an individual has, the higher the cognitive strain, consequently reducing acceptability. Too much freedom of choice was determined to be more burdensome, leading to lower levels of engagement compared to coherently structured intervention components ( Schwartz, 2000; Ware *et al*., 2012). It can be argued that SMART has a sufficient balance of progression and coherent intervention components.

### Strengths and limitations

This study identified that the SMART intervention has satisfactory usability and acceptability for MS patients, whilst also suggesting future adaptations to improve accessibility for this population (particularly if seeking to make the intervention widely available in future practice, contingent on efficacy testing). The SMART system was tested on more women than men representing the higher prevalence of MS in women that we see in the general population ( Harbo *et al*., 2013). There are, however, several limitations. The participant sample consisted of MS sufferers with mixed cognitive abilities, according to the SDMT. Most participants (58%) performed in the average range of cognitive functioning, whereas the prospective feasibility trial (and potential future applications of SMART) will be with people experiencing cognitive impairment. Therefore, it is unknown how transferable the results are to the most cognitively impaired. However, our sample was inclusive of five participants with impaired performance on the SDMT, and these individuals were still able to engage with the interface successfully, indicating usability in the target population. It should also be noted that, even where SDMT scores are consistent with normative averages, this may still represent an individual-level decline from pre-diagnosis performance ( Healy *et al*., 2021).

### Implications and future research

This prefeasibility study informs potentially impactful research for MS patients, with implications for future clinical practice. SMART is shown to be testable and has a satisfactory level of usability and acceptability within the MS population. These findings provide evidence to suggest a feasibility trial is an appropriate next step: building on evidence for acceptability to inform potential progression to a randomised control trial.

Future research should consider specific adaptations to make the mobile phone formatting more user-friendly for MS participants. It may be helpful to enable participants to log in with a familiar username and password to account for memory deficits in the MS population, and to reduce cognitive strain and unnecessary burden. To potentiate engagement, it may be advantageous to (1) introduce more elements to break up the repetitive nature of the levels and (2) provide leeway in progression requirements for participants with MS, to account for accidental errors ( *e.g.*, due to tremors and hand-eye coordination). However, such changes may have an impact on targeted learning (progressive development of relational abilities) so would warrant careful pretesting.

### Conclusions

With facilitator support, the SMART intervention is fit for purpose for progression to a feasibility trial, with the intentions of developing a randomised controlled trial of its efficacy for improving cognitive difficulties in people with MS.

## Data availability

### Underlying data

The qualitative dataset is not publicly available due to greater potential identifiability and because participants only consented to sharing of anonymised transcripts with other researchers (rather than as open data per se). Reasonable requests from researchers for access to anonymised qualitative data will be considered. Please contact the corresponding author via email: Alexandra.frost@nottshc.nhs.uk.

The anonymised quantitative dataset for this study, representing sample characteristics and cognitive assessment results, is publicly available via a repository:

Open Science Framework: SMART MS prefeasibility evaluation. https://doi.org/10.17605/OSF.IO/CP6AH ( Frost *et al*., 2022).

This project contains the following underlying data:

- SMART MS prefeasibility evaluation data.sav

Data are available under the terms of the Creative Commons Attribution 4.0 International license (CC-BY 4.0).

### Extended data

Open Science Framework: SMART MS prefeasibility evaluation. https://doi.org/10.17605/OSF.IO/CP6AH ( Frost *et al*., 2022).

This project contains the following extended data:

- SMART MS Cognitive Interview Guide v1.0 050521.docx
- COREQ.docx
- KEY (ngm).docx
- SMART MS Consent Form (Alpha Testing) v2.0 040621.doc
- SMART MS Consent Form (Cognitive Interview) v2.0 040621.doc
- SMART MS Description of SMART Programme v1.0 050521.docx
- SMART MS Participant Information Sheet (Alpha Testing) v2.0 040621.docx
- SMART MS Participant Information Sheet (Cognitive Interview) v2.0 040621.docx
- SMART MS Questionnaire v1.0 040621 (1).docx
- SMART MS Alpha-testing Feedback Interview Schedule v1.0 050521.docx

Data are available under the terms of the Creative Commons Attribution 4.0 International license (CC-BY 4.0).

## Research team and reflexivity

### Personal characteristics

Miss Alexandra Frost (She/Her - BSc, MSc, PhD in progress) conducted both the ‘think-aloud’ protocol and the alpha-testing phase of research. At the time of the study Miss Frost was a research assistant on the SMART MS prefeasibility project. Miss Frost has experience of recruitment and research stemming from her position as a PhD researcher.

***Relationship with participants.*** Miss Frost aimed to gain rapport with potential participants before and during the study. One participant was previously known to Miss Frost. This meant there was less need to establish rapport prior to the study commencing. Miss Frost took care to consider how the existing relationship might impact the interview itself, therefore an interview schedule was used in a standardised way in order to ensure that the same topics were addressed for every participant. Participants were informed of the aims of the research prior to participation. Miss Frost has interests in the research areas of MS and cognitive functioning. Miss Frost had no previous experience of using the SMART programme and had no part in its development.
