# Supplementary material for: Usability and acceptability of a cognitive training intervention (SMART) for people with multiple sclerosis (MS): A prefeasibility formative evaluation
Source: NIHR Open Res. 2022 May 23;2:39. [Version 1] doi: 10.3310/nihropenres.13274.1 (PMC10593333; doi:10.3310/nihropenres.13274.1)
Supplement: Peer review history

# Reviewer report: Reviewer response for version 1

**DOI:** 10.3310/nihropenres.14393.r30121

**Published:** 2023-08-17

**Authors:** Jessica Podda

**Affiliations:** 1 Italian Multiple Sclerosis Foundation, Genoa, Italy

The paper titled "Usability and acceptability of a cognitive training intervention (SMART) for people with multiple sclerosis (MS): A prefeasibility formative evaluation" aimed to test the usability and acceptability of a computerized cognitive training intervention named Strengthening Mental Abilities Through Relational Training (SMART) for people with MS, using a mixed-method design.

 One of the biggest challenge for implementing novel technologies into clinical practice is providing detailed validation and usability testing procedures. Thus, authors' attempt is highly appreciated. Considering the success of tele-medicine highlighted during COVID-19 global pandemic, I found this research very promising and interesting. One of the main strengths of the paper is such a fine-grained think-aloud process. The 12 concurrent ‘think-aloud’ feedback prompts and six retrospective prompts are proper to investigate usability and acceptability of a novel digital system. 

 Although, I have minor comments:

 Considering such rigorous protocol performed remotely, I'm afraid that people with lower digital competencies would have not be encouraged to take part to the study. For instance, older people may not be familiar with Microsoft Teams call. I was wondering whether participants were autonomous throughout the task completion or they could ask to a caregivers for a help. Maybe, investigating how people are familiar with technology using a specific questionnaire could be interesting to draw conclusion about the acceptability of the computerized system. 

 Furthermore, I do not clearly understand the assessment administration. How did people with MS perform the SDMT? Alone or with the clinician remotely? I suggest to better describe this aspect. This could improve clarity of the study.

Is the work clearly and accurately presented and does it cite the current literature?

Yes

If applicable, is the statistical analysis and its interpretation appropriate?

Not applicable

Are all the source data underlying the results available to ensure full reproducibility?

Yes

Is the study design appropriate and is the work technically sound?

Yes

Are the conclusions drawn adequately supported by the results?

Yes

Are sufficient details of methods and analysis provided to allow replication by others?

Yes

Reviewer Expertise:

Cognition and Technology in MS

I confirm that I have read this submission and believe that I have an appropriate level of expertise to confirm that it is of an acceptable scientific standard.

# Reviewer report: Reviewer response for version 1

**DOI:** 10.3310/nihropenres.14393.r29410

**Published:** 2023-06-14

**Authors:** Christopher P. Dwyer

**Affiliations:** 1 Applied Psychology, School of Social Science, Technological University of the Shannon Midlands Midwest, Limerick, County Limerick, Ireland

This research was a prefeasibility assessment of the usability and acceptability of a computerised cognitive training intervention—Strengthening Mental Abilities Through Relational Training (SMART) —for people with MS. Specifically, twelve people with MS were involved in a think-aloud protocol ( *n *= 6) to assess initial usability and alpha-testing ( *n *= 6) to assess acceptability. Results suggested satisfactory usability and acceptability. Minor suggestions were made for optimising SMART for people with MS. The authors recommend progression to a feasibility trial of SMART. 

 Minor point, but I think the introduction would benefit with an extra line or two explaining the rationale for computerised cognitive training. I’m aware that a slew of references have been provided, but I think a brief, explicit statement of reasoning will help the flow.

 “SMART was developed from a well-established theory of cognition and language” – provide reference(s)…I see you address this later, but including them front and centre will provide greater clarity.

 I notice in the criteria that level of cognitive difficulty is not addressed. This raises questions for me...Is this training a preventative measure against possible cognitive difficulty in the future, is it a rehabilitation programme or something else? I ask because those with/without cognitive difficulty might skew data one way or the other depending on the purpose of this training. This is by no means a damning issue, just a point that requires greater clarity. Alternatively, I understand that this is prefeasibility and the authors might want to be assessing all scenarios, which is also fine. With that, the aims regarding prefeasibility assessment may benefit from more emphasis than just a mention in the introductory section. Again, this is all just an issue of clarity.

 Overall, this is a very interesting study and design and I look forward to seeing the feasibility trial.

Is the work clearly and accurately presented and does it cite the current literature?

Yes

If applicable, is the statistical analysis and its interpretation appropriate?

Not applicable

Are all the source data underlying the results available to ensure full reproducibility?

Yes

Is the study design appropriate and is the work technically sound?

Yes

Are the conclusions drawn adequately supported by the results?

Yes

Are sufficient details of methods and analysis provided to allow replication by others?

Yes

Reviewer Expertise:

Cognition in MS

I confirm that I have read this submission and believe that I have an appropriate level of expertise to confirm that it is of an acceptable scientific standard.
